# Assessment of ST2 and Reg3a levels in patients with acute graft-versus-host disease after allogeneic hematopoietic stem cell transplantation

**DOI:** 10.3906/sag-2007-17

**Published:** 2021-02-26

**Authors:** Elifcan ALADAĞ, Haluk DEMİROĞLU, Ümit Yavuz MALKAN, Ümit AKMAN, Hakan GÖKER, Yahya BÜYÜKAŞIK

**Affiliations:** 1 Department of Hematology, Faculty of Medicine, Hacettepe University, Ankara Turkey; 2 Department of Hematology, Dışkapı Yıldırım Beyazıt Training and Research Hospital, University of Health Sciences, Ankara Turkey

**Keywords:** Suppression of tumorigenicity 2, regenerating isletderived 3-alpha, graft-versus-host disease, allogeneic hematopoietic stem cell transplantation

## Abstract

**Background/aim:**

Graft-versus-host disease (GVHD) is a crucial complication leading to significant morbidity and mortality allogeneic hematopoietic stem cell transplantation which occurs in approximately half of the transplant recipients. Suppression of tumorigenicity 2 (ST2) and regenerating islet-derived 3-alpha(Reg3a) might be important biomarkers to predict acute GVHD.

**Materials and methods:**

In the present study, blood samples were collected from 17 patients with acute GVHD and 12 control patients after allogeneic stem cell transplantation. ST2 and Reg3a were measured in plasma samples compared in patients with acute GVHD and the controls.

**Results:**

Median age of the study population was 42 years (range 19–49). When compared to controls, the mean ST2 levels was significant higher in acute GVHD (9794 ng/dL vs. 2646 ng/dL, P = 0.008). Mean Reg3a level did not show significant difference between control and acute GVHD group (8848 ng/dL vs. 5632 ng/dL, respectively, P = 0.190).

**Conclusion:**

The ST2 level might be used as a significant biomarker for predicting acute GVHD.

## 1. Introduction

Acute graft versus host disease (GVHD) is an important cause for morbidity and non-relapse mortality observed in nearly half of allogeneic hematopoietic stem cell transplantation (AHSCT) patients [1–3]. In recent years, the improvements on the infection prophylaxis and monitoring, immunosuppressive strategies and advances in supportive care have improved outcomes after AHSCT. Although acute GVHD might affect all organs, it is most commonly observed in skin, liver, and gastrointestinal tract and typically occurs at the 100th day of AHSCT [4]. High-dose systemic glucocorticoids remain the first-line treatment for acute GVHD. Complete response is achieved within a month in approximately half of patients under steroid treatment [5]. Resistance to steroid therapy is an important predictor of non-relapse mortality and patients have a very high mortality rate within 6 months [6]. Limited progress has been made in the treatment of GVHD, highlighting the importance of developing more effective prediction and prevention strategies. Several clinical, genetic, and biomarker-based risk factors have been identified that correlate with acute GVHD risk [7]. The aim of the present study was to investigate the potential role of two markers, Suppression of tumorigenicity 2 (ST2) and Regenerating islet-derived protein 3 alpha (Reg3a), in the prediction of acute GVHD. 

## 2. Subjects and methods

### 2.1. Study population

The present study was performed in the Bone Marrow Transplantation Unit of Hacettepe University Haematology Department. Plasma samples were collected from 17 patients with acute GVHD diagnosed tissue biopsy receiving transplantation for acute leukemia. Similarly, as the control group, plasma samples of 12 patients who were followed-up after transplantation were randomly collected. 

### 2.2. Plasma samples collection 

Phlebotomists drew whole blood into lavender tubes (treated with a K2EDTA; BD Diagnostics Ref368499) after GVHD had occurred. Plasma was isolated by centrifugation for 15 min at 2000xg (NUVE NF 400R; Nuve, Turkey) at 4oC. Centrifugation time after blood collection was within 30 minute. Supernatant (Plasma) was removed using a Pasteurpipette and obtained plasma sample aliquot into to eppendorph tube it was divided into aliquot (Eppendorf tub) and immediately frozen at ultra low temperature deep freeze (U410 New Brunswick Scientific, Eppendorf North America Inc., Framingham, MA, USA) and storied at –80 oC until processing.

ST2 and Reg3A levels were measured by sandwich ELISA. Human IL-1 RL1 /ST2 Pico Kine ELISA was purchased from Boster Biological Technology Ltd. (Catalogue Number: EK1116; Pleasanton, CA, ABD). Human Reg3A Duo set Elisa was purchased from R&D Systems Inc. (Catalogue Number: DY5940-05; Minneapolis, MN, USA & Canada). ST2 and Reg3A were measured according to manufacturer’s instruction in prospectus. Samples were diluted 1:5 for ST2 and 1:51 for Reg3A. We analysed these samples and standards in single using a Sunrise microplate reader (Tecan Group AG, Männedorf, Switzerland). Absorbance’s results of measurement for Reg3A and ST2 were calculated with Magellan Tracker V6.5 Data Analysis Software (Tecan Group AG) 

### 2.3. Statistical analysis

Statistical analysis was performed using SPSS software (version 20.0; IBM Corp., Armonk, NY, USA). Between-group differences were assessed with the use of either a chi-square test or a Wilcoxon rank-sum test. Cox regression, and competing-risks regression were used to evaluate the association of the six-biomarker panel. Competing models were compared with the use of two metrics: hazard ratios and their 95% confidence intervals.

## 3. Results

The study included 17 patients with acute GVHD and 12 controls who were followed up after AHSCT. The main demographic and clinical characteristic of patients are shown in Table. Median follow-up was 19.5 months (range 4–92) and median follow-up of the patients after acute GVHD diagnosis was 4.9 months (range 0.46–37.5). Patients with acute GVHD had eye, skin, and liver involvement. In addition, 8 patients had lower GIS GVHD.

**Table:** The main demographic and clinical characteristics of the study population.

Characteristics	Acute GVHD patients(n = 17)	Control(n = 12)
Median age, patients (years)	38.3 [19 – 47]	34.0 [21 – 58]
Median age, donor (years)	32.5 [18 – 52]	40.0 [14 – 58]
Sex (F/M) (%)	7/10 (41.2/58.8)	4/8 (33.3/66.7)
Donor sex mismatch	8 (47.1)	5 (41.7)
Diagnosis Acute myeloid leukemia Acute lymphoid leukemiaOther	764	921
Conditioning regimens Myeloablative Reduced intensity	116	93
Median infused MNC dose (×108)	5.6 [4.1–13.1]	5.6 [2.1–6.7]
Median infused CD34 dose (×106/kg)	8.8 [3–28.2]	8.7 [4.7–20.8]
GVHD prophylaxisMtx-CNIMtx-CNI-ATGCNI-MMF	773	561

ATG: antithymocyte globulin; CNI: calcineurin inhibitor; F: female; GVHD: graft-versus-host disease; M: male; MNC: mononuclear cell; MMF: mycophenolate mofetil; Mtx: methotrexate. Numerical variables were expressed as median (minimum-maximum).

Mean ST2 level was found to be significantly higher in patients with acute GVHD compared to those without acute GVHD (9794 ng/dL vs. 2646 ng/dL respectively, P = 0.008) (Figure A). On the contrary, mean Reg3a level did not show significant difference between acute GVHD and control group (8848 ng/dL vs 5632 ng/dL respectively, P = 0.190) (Figure B). Nevertheless, plasma Reg3a levels was found to be higher in patients with GIS GVHD than those with other GVHD, but this difference did not reach statistical significance (11850 ng/dL vs. 6180 ng/dL, P = 0.132).

**Figure:**
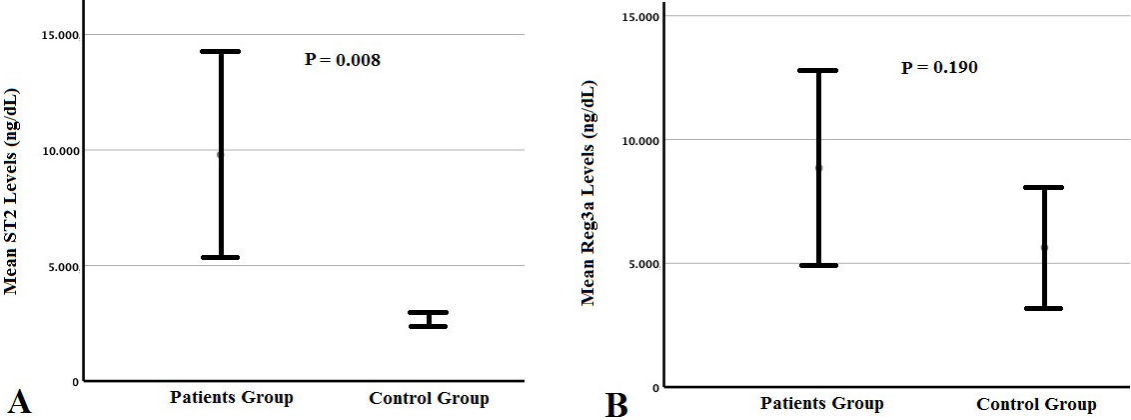
Box-plot graphs of the patients’ and controls’ ST2 (A) and Reg3a (B) levels.

## 4. Discussion

In the present study, ST2 level was found to be higher in patients with acute GVHD compared to control but Reg3 level was comparable. ST2 and Reg3a have been shown to predict GVHD development and nonrelapse mortality in patients of acute HSCT in the previous studies [8, 9]. In a study using this 2 biomarker with severe GVHD by Hartwell and colleagues, they showed that patients with high ST2 and Reg3 levels had higher 6-month mortality, and that the combination of these two biomarkers could be a predictive test for nonrelapse mortality [10]. 

Soluble ST2 acts as a decoy receptor for interleukin-33 and drives T helper 2 cells toward a type 1 helper T cell phenotype, which may be important in the pathophysiology of GVHD.It is arguable that ST2 monitoring in AHSCT might identify these patients and prevent them from developing acute GVHD.

The findings of a study performed by Vander Lugt et al. are consistent with the present study [11]. The authors have found that ST2 had the most significant association with resistance to GVHD therapy and nonrelapse mortality. Patients with high ST2 levels, treatment-resistant GVHD development was 2.5 times higher than in patients with low ST2 levels.

Regenerating islet-derived protein 3 alpha (Reg3A) is a biomarker of lower gastrointestinal GVHD; however, the biological role of Reg3A in the pathophysiology of GVHD is not well understood [9]. In a study conducted by Ferrara et al. by 162 patients with low gastrointestinal system GVHD, Reg3a level was found to be 4-fold higher than in the control group [9]. Similarly, in the present study, although Reg3a level was similar inpatients with and without GVHD, patients with GIS GVHD levels were higher 2 times in those with other GVHD. However, the difference did not reach statistical significance. This result can be explained by the small number of patients.

This study has some limitations. Firstly the number of patients is less. Since median follow-up was short a survival association with biomarker levels could not be evaluated. Another limitation of this study is that patients do not have basal biomarker levels before the development of GVHD after AHSCT. Comparing the increase after GVHD with baseline values of the patients would be more accurate approach to demonstrate the predictive power of this biomarker.

## 5. Conclusion

The ST2 level might be used as a significant biomarker for predicting acute GVHD. However, further studies with larger patient populations are needed for clinical use. 

## Informed consent

As a standard of care/action of the hospitals of Hacettepe Medical School, all of the ethical considerations were strictly followed, all of the patients gave informed consent for the procedure at the time of hospitalization and before the administration of chemotherapy and other relevant diagnostic/therapeutic procedures, in accordance with the Declaration of Helsinki. This study is approved by our University Ethical Board on 06.07.2018 with the approval number of GO 18/490-14.
